# Identification of a Linear B-Cell Epitope in the African Swine Fever Virus pE248R Protein Targeted by Monoclonal Antibodies

**DOI:** 10.3390/microorganisms13112616

**Published:** 2025-11-18

**Authors:** Enping Liu, Xinyue Liu, Yumei Chen, Hongliang Liu, Jingming Zhou, Aiping Wang

**Affiliations:** 1 School of Life Sciences, Zhengzhou University, Zhengzhou 450001, China; 2 Henan Key Laboratory of Immunobiology, Zhengzhou 450001, China; 3 Longhu Laboratory, Zhengzhou 450002, China

**Keywords:** ASFV, pE248R protein, monoclonal antibodies, B-cell epitope

## Abstract

African swine fever virus (ASFV) is the only member of the family Asfarviridae and can cause African swine fever, a disease with a consistently high mortality rate. The pE248R protein, a myristoylated integral membrane protein of ASFV, is required for virus infectivity and some early postentry event, making it a key target for studying the prevention and treatment of ASFV. In this study, BALB/c mice were immunized with purified recombinant pE248R protein, leading to the generation of five monoclonal antibodies (mAbs). Selected mAbs were subsequently subjected to further characterization. By identifying the reactivity of different pE248R protein peptide segments with these monoclonal antibodies, we screened and identified a linear B cell epitope (^87^QEVALTQWMDAG^98^) on the pE248R protein. These results provide a new theoretical basis for analyzing the structure and function of pE248R protein, particularly contributing to the construction of a comprehensive B-cell epitope map for ASFV immunogens.

## 1. Introduction

African swine fever (ASF) is a highly contagious viral disease caused by the African swine fever virus (ASFV), with a mortality rate of up to 100% in clinically infected pigs, leading to significant economic losses in the swine industry [1,2].

ASFV is a large, enveloped, double-stranded DNA virus and the sole member of the family Asfarviridae [3]. It is also the only known arthropod-borne DNA virus (DNA arbovirus) identified to date [4]. The virion displays an icosahedral, multilayered architecture with an average diameter of approximately 200 nm [5,6]. Its core comprises a nucleoid housing the viral genome and associated nucleoproteins, which is sequentially enclosed by a core shell, an inner envelope, an icosahedral capsid, and an outer envelope [7]. The ASFV genome is a linear double-stranded DNA molecule, 170–194 kilobases (kb) in length, characterized by inverted terminal repeats and hairpin loops at both ends. It contains more than 150 open reading frames (ORFs) [4] encoding a wide variety of proteins—over 60 structural and more than 100 nonstructural proteins [8]. Following cellular entry, the viral genome is delivered into the cytoplasm, where transcription of early viral genes is initiated [9].

Linear B-cell epitopes can activate specific humoral immune responses in the host. Characterized by continuous amino acid sequences, these epitopes can be readily identified in vitro and are commonly utilized in disease diagnosis, vaccine design, and antibody production [10]. Given that humoral immunity provides partial protection against ASFV infection, the identification of B-cell epitopes in viral proteins is crucial for the development of ASFV subunit vaccines [11]. In recent years, B-cell epitopes of various ASFV proteins—including p72, p54, p30, pH108R, p17, pE120R, and pE199L—have been extensively identified [12,13,14,15]. However, due to the diversity and structural complexity of viral proteins, no effective protective vaccine has been developed thus far [16]. Therefore, further investigation into the B-cell epitopes of ASFV proteins remains imperative.

The pE248R protein is a structural component of the mature ASFV virion and functions as a substrate in viral-encoded redox reactions, relying on disulfide bonds for its correct folding and assembly [17]. Its amino acid sequence contains an N-terminal myristoylation site and a C-terminal hydrophobic transmembrane domain [18]. Functionally, pE248R is critical during the early phase of viral infection. The absence of pE248R markedly impairs both early and late viral gene expression, reducing ASFV infectivity by approximately 100-fold. Although not essential for virion assembly, pE248R may contribute to virion maturation and modification, as well as to the facilitation of viral entry into host cells [19]. Hernáez et al. demonstrated that the ASFV pE248R protein is essential for virus-cell fusion but dispensable for viral uncoating, and it promotes membrane fusion and subsequent release of the viral core into the cytoplasm [20]. Furthermore, pE248R significantly enhances ASFV replication in vitro, underscoring its crucial role in viral propagation [21]. Regarding host immune responses, studies have revealed that the pE248R protein suppresses interferon production by modulating both the cGAS-STING and RIG-I signaling pathways, thereby facilitating viral evasion of host immune surveillance [22,23]. This immunomodulatory function underscores the role of pE248R in counteracting the host’s antiviral defense. Notably, pE248R shares amino acid sequence homology with the L1 protein of vaccinia virus (VACV), which is part of the multiprotein entry-fusion complex required for membrane fusion or core penetration [24,25]. The VACV L1 protein is also a major target of neutralizing antibodies [26]. In a recent vaccine development study, a chimeric immunogen was constructed by fusing the N-terminal 198-amino-acid fragment of pE248R with other ASFV proteins or epitopes. This vaccine candidate successfully induced robust ASFV-specific humoral and cellular immunity in pigs [27]. In summary, the pE248R protein plays an essential role in ASFV replication, infection, immune evasion and the E248R gene may represent a promising candidate target for the development of vaccines or therapeutics against ASFV infection.

In this study, the E248R gene was cloned into a pET-28a vector, and the recombinant plasmid was transformed into *Escherichia coli* for induced protein expression. Through immunization of mice with the purified recombinant protein and subsequent hybridoma screening, five specific mAbs were obtained. Using the reactivity profiles of these mAbs against a series of truncated pE248R peptides, a linear B-cell epitope, ^87^QEVALTQWMDAG^98^, was identified. These findings provide a theoretical basis for the further functional characterization of the pE248R protein while also enriching the B-cell epitope map of ASFV immunogens.

## 2. Materials and Methods

### 2.1. Plasmids

The pET-28a and pET-32a plasmids were obtained from the Molecular Immunology Laboratory at Zhengzhou University. The coding sequence of ASFV E248R (GenBank Accession No. AYW34102.1) was codon-optimized and synthesized by Sangon Biotech (Shanghai, China). The E248R gene was cloned into the pET-28a and pET-32a vectors using a restriction enzyme-based digestion method. DNA fragments P1–P4 and P2-1~P2-4 were amplified by PCR using the PrimeSTAR DNA Polymerase Kit (TaKaRa, Shanghai, China). The specific primers used for amplifying these fragments are listed in Table 1.

### 2.2. Purification of pE248R Protein

Upon reaching an OD_600_ of 0.6–0.8, the recombinant bacterial culture expressing pE248R was induced with 0.2 mM IPTG for 18 h. Cells were then harvested by centrifugation, resuspended in Tris-HCl-NaCl buffer (pH 7.5), and lysed by ultrasonication. The lysate was centrifuged at 12,000× *g* for 15 min at 4 °C to separate the soluble and insoluble fractions. The pellet, containing inclusion bodies, was washed three times with washing buffer (20 mM Tris-HCl, 150 mM NaCl, 2 M urea, pH 7.5) and recovered by centrifugation after each wash. The washed inclusion bodies were solubilized in denaturation buffer (20 mM Tris-HCl, 150 mM NaCl, 8 M urea, pH 7.5) with continuous stirring until fully dissolved. The solubilized sample was clarified by centrifugation and filtration, then loaded into a dialysis membrane. Stepwise dialysis was performed at 4 °C against renaturation buffer to refold the protein. The refolded protein solution was filtered and further purified by nickel affinity chromatography. Protein expression and identity were confirmed by SDS-PAGE and Western blot analysis.

### 2.3. SDS-PAGE and Western Blot

The SDS-PAGE was performed as follows: the target protein was resuspended in 1× protein loading buffer [0.2 M Tris-HCl pH 6.8; 5% sodium dodecyl sulfate (*w*/*v*); 2% bromophenol blue (*w*/*v*); 0.1% glycerol (*w*/*v*); 2% 5 M DTT (*v*/*v*)], heated at 98 °C in a metal bath for 15 min, centrifuged at 1000× *g* for 30 s, and then 20 μL (200 ng) was loaded onto an SDS-PAGE gel [(10% polyacrylamide resolving gel (lower gel) was overlaid with a 4% polyacrylamide stacking gel (upper gel)] for electrophoresis (80 V/20 min and 120 V/100 min).

The identification of pE248R protein was assessed via Western blot. The Western blot assay was performed as described previously [28] and primary antibody was monoclonal anti-His antibody (Solarbio, Beijing, China) or ASFV-positive swine serum (China Veterinary Drug Inspection Center).

### 2.4. Cell Culture and Animals

Competent *Escherichia coli* cells (DH5α and BL21 strains) were purchased from Weidi Biotechnology (Shanghai, China). Human Embryonic Kidney cells (HEK293T cells) were obtained from the Shanghai Cell Bank of the Chinese Academy of Sciences.

For the generation of positive hybridoma cells, two healthy BALB/c mice (6–8 weeks old), sourced from the Experimental Animal Center of Zhengzhou University, were immunized with recombinant pE248R protein emulsified in complete Freund’s adjuvant. The mouse exhibiting the highest serum antibody titer after three immunizations was selected for a final booster injection. Three days later, splenocytes from the immunized mouse were fused with SP2/0 myeloma cells using polyethylene glycol. After seven days of culture, hybridoma supernatants were screened by indirect ELISA to identify clones stably secreting antigen-specific monoclonal antibodies. Positive hybridoma cells were subsequently subcloned by the limiting dilution method to ensure monoclonality [29].

### 2.5. Indirect Enzyme-Linked Immunosorbent Assay (iELISA)

The antibody titer in mouse serum and the identification of positive hybridoma clones were evaluated by iELISA. Purified pE248R protein diluted in carbonate-bicarbonate buffer (CBS) was coated onto 96-well microplates and incubated at 37 °C for 2 h. After washing with Phosphate-Buffered Saline with Tween^®^ 20 (PBST), the plates were blocked with a blocking buffer for 2 h at 37 °C. Subsequently, immune serum or hybridoma supernatant was added as the primary antibody and incubated at 37 °C for 1 h. Following four washes with PBST, the plates were incubated with HRP-conjugated goat anti-mouse IgG for 1 h at 37 °C and washed again four times. Color development was initiated by adding TMB substrate solution and allowed to proceed for 5 min before the reaction was terminated with 2 M H_2_SO_4_. Absorbance was measured at 450 nm using a microplate reader (Bio-Rad, Hercules, CA, USA).

### 2.6. Purification and Characterization of Monoclonal Antibodies

Monoclonal antibody ascites was produced in female mice by intraperitoneal inoculation of positive hybridoma cells and initially characterized by SDS-PAGE. The titer and affinity of mAbs against pE248R protein were evaluated using iELISA. A saturation curve was generated by plotting the monoclonal antibody concentration against the corresponding OD_450_ value, with the maximum OD_450_ value defined as 100% binding.

For affinity determination, a double-reciprocal plot was constructed using the inverse of the monoclonal antibody concentration versus the inverse of the OD_450_ value. The molar concentration of monoclonal antibody yielding 50% of the maximum OD_450_ value was determined. The affinity constant (Kaff) was calculated according to the following formula [30]:

Kaff = (n − 1)/2(n[Ab’]_t_ − [Ab]_t_)

where n represents the ratio of antigen concentrations [Ag]_t_ to [Ag’]_t_, with [Ag]_t_ = 2 μg/mL and [Ag’]_t_ = 1 μg/mL of pE248R protein. [Ab]_t_ and [Ab’]_t_ denote the molar concentrations of monoclonal antibody required to achieve 50% of the maximum OD_450_ value at the corresponding antigen concentrations.

### 2.7. Immunofluorescence Assay (IFA)

IFA was performed to evaluate the reactivity and specificity of the screened anti-pE248R monoclonal antibodies with recombinant ASFV protein pE248R expressed in the eukaryotic expression system. Eukaryotic cells HEK293T were revived and cultured until reaching 40–50% confluence, then transfected with the pCAGGS-pE248R plasmid. Forty-eight hours post-transfection, the cells were fixed with 4% paraformaldehyde. After fixation and washing, the cells were incubated with anti-pE248R mAbs for 1 h, followed by three washes with Phosphate-buffered saline (PBS). Subsequently, the cells were incubated with Cy3-conjugated goat anti-mouse IgG (Bioss, Beijing, China) for 1 h. Nuclei were counterstained with DAPI (Solarbio, Beijing, China), and fluorescence signals were visualized using an inverted fluorescence microscope (Olympus, Tokyo, Japan).

### 2.8. Peptide Synthesis and Epitope Mapping

To precisely define the linear B-cell epitope recognized by the monoclonal antibody, a set of consecutive overlapping peptides spanning residues 80–105 of the pE248R protein were designed and synthesized in GL Biochem (Shanghai) Ltd. (Shanghai, China). (Table 2). These peptides were conjugated to a carrier protein using the glutaraldehyde method [31]. The immunoreactivity of the identified epitope peptide was subsequently confirmed with ASFV-positive swine serum using both peptide ELISA and dot-blot assays.

### 2.9. Peptide-ELISA and Dot-Blot

The peptide-ELISA was performed by coating the synthetic peptide onto 96-well plates at a concentration of 5 µg/well using CBS. Subsequent steps, including blocking, incubation with antibodies, and signal detection, were carried out as described for the iELISA protocol. PBS and recombinant pE248R protein were used as the negative and positive controls, respectively.

For the dot-blot assay, 2 µL of peptide solution (4 mg/mL) was directly spotted onto a nitrocellulose membrane that had been pre-equilibrated with PBS. After air-drying, the membrane was blocked with 5% skimmed milk at 37 °C and then probed with the anti-pE248R monoclonal antibody as the primary antibody. All subsequent washing, secondary antibody incubation, and detection steps were performed according to standard Western blot procedures.

### 2.10. Conservation Analysis and Spatial Localization of the Epitope

The conservation of amino acid sequences was analyzed using MUSCLE alignment (Version 3.8.21) in the Jalview software (Version 2.11.5.0). The 3D structure of the ASFV pE248R protein was modeled using homology modeling through the SWISS-MODEL (https://swissmodel.expasy.org/, accessed on 11 August 2025). The epitope sequence recognized by mAb was visualized on the pE248R protein using PyMOL software (Version 3.1.3.1).

## 3. Results

### 3.1. Expression of Recombinant pE248R Protein

**Figure 1:**
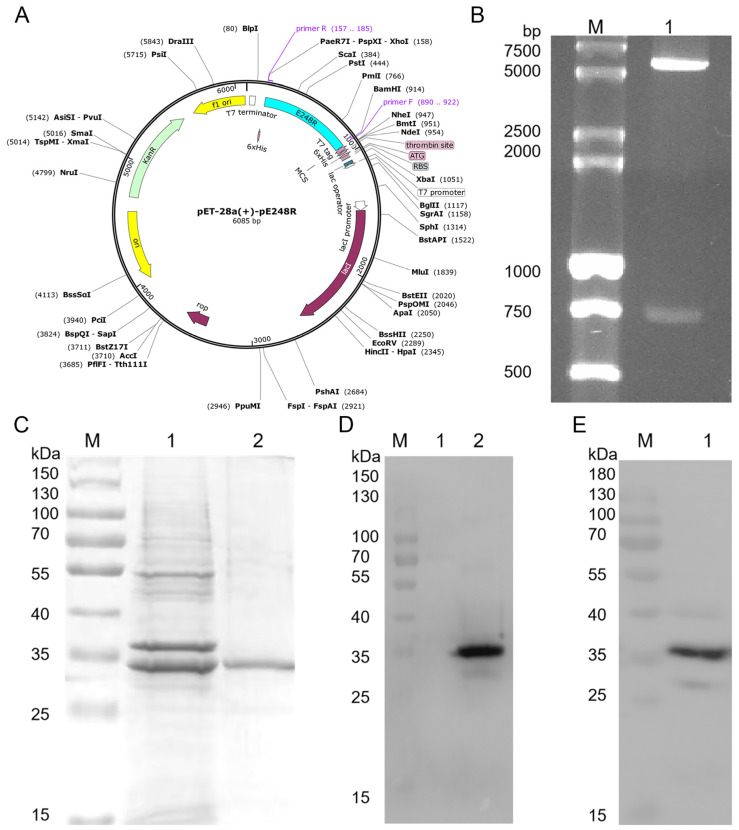
Construction and induced expression of recombinant expression plasmids. (**A**) Schematic of pET-28a(+)-pE248R vector construction. (**B**) Double enzyme digestion verification. Lane M, DL 15,000 + 2000 DNA marker. Lane 1, pET-28a-pE248R double enzyme digestion product. (**C**) SDS-PAGE identification of purified pE248R. Lane M, protein marker. Lane 1, unpurified pE248R recombinant protein. Lane 2, purified pE248R protein. (**D**) Western blot identification of pE248R using anti-His monoclonal antibodies. Lane M, protein marker. Lane 1, the pET-28a vector protein. Lane 2, pE248R recombinant protein. (**E**) Western blot identification of pE248R using ASFV-positive swine serum. Lane M, protein marker. Lane 1, pE248R protein.

The construction map of the pET-28a-E248R recombinant plasmid is Figure 1A. Following transformation into *Escherichia coli* and amplification, the plasmid was extracted and verified by double restriction enzyme digestion (Figure 1B), confirming the successful construction of the pET-28a-pE248R expression vector. The recombinant pE248R protein, with an apparent molecular weight of approximately 35 kDa, was predominantly expressed in inclusion bodies. After optimizing the method and concentrating, a highly purified recombinant protein was obtained (Figure 1C). The concentration of the purified pE248R protein was measured as 0.3 mg/mL using UV spectrophotometry. Western blot analysis demonstrated that the recombinant pE248R protein specifically reacted with both ASFV-positive swine serum and a monoclonal anti-His antibody (Figure 1D,E). These results confirm the successful expression and purification of the pE248R recombinant protein, supporting its suitability for subsequent animal immunization.

### 3.2. Screening of mAbs Against the ASFV pE248R Protein

**Figure 2:**
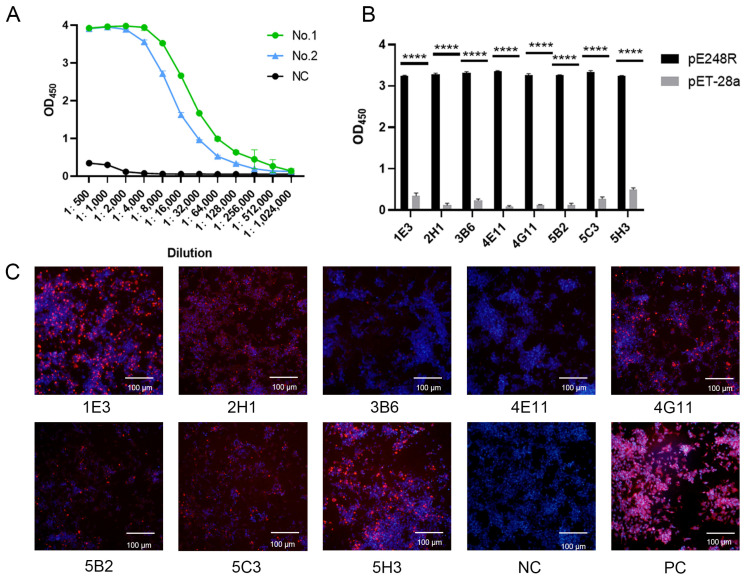
Screening of mAbs against the ASFV pE248R protein. (**A**) Titers of the pE248R-specific immune serum. Unimmunized mouse serum was used as a negative control. (**B**) Selection of eight positive hybridoma clones. (**C**) IFA analysis of the reactivity between the eight mAbs and the pE248R protein. Red fluorescence indicates anti-pE248R mAbs, while blue fluorescence represents the nuclei. The negative control was PBS. Scale bar, 100 μm. Data shown in B are representative of three independent experiments (mean + SD of duplicate experiments). **** *p* < 0.0001 versus the control groups; and Student’s *t*-test.

The serum antibody titers specific to the pE248R protein were quantified by iELISA. Seven days after the booster immunization, antibody titers in mice reached levels ranging from 1:2.56 × 10^5^ to 1:5.12 × 10^5^ (Figure 2A), indicating a robust humoral immune response following the three immunizations. Following cell fusion and subcloning, eight distinct monoclonal hybridoma lines—designated 1E3, 2H1, 3B6, 4E11, 4G11, 5B2, 5C3, and 5H3—were successfully established (Figure 2B). IFA was performed to assess the reactivity of the resulting monoclonal antibodies with eukaryotic-expressed pE248R in HEK293T cells (Figure 2C). The results showed that clones 1E3, 2H1, 4G11, 5C3, and 5H3 exhibited strong and specific binding to the recombinant pE248R protein.

### 3.3. Purification and Characterization of mAbs

**Figure 3:**
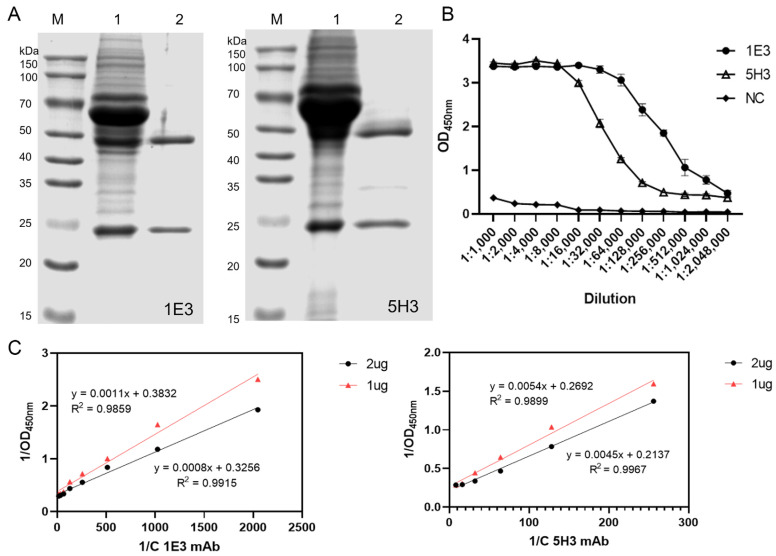
Purification and characterization of mAbs. (**A**) Identification of the purified mAbs. Lane M: protein marker; Lane 1: before purification; Lane 2: after purification. (**B**) Determination of the titer of the monoclonal antibodies. (**C**) Affinity analysis of the two monoclonal antibodies.

Two hybridoma clones, 1E3 and 5H3, were selected for ascites production in female BALB/c mice. Analysis of the ascitic fluid by SDS-PAGE before and after purification confirmed the high purity of the resulting monoclonal antibodies (Figure 3A). The titers of the purified mAbs, as determined by iELISA, reached 1:2.048 × 10^6^ for both 1E3 and 5H3 (Figure 3B). Affinity measurements yielded affinity constants of 1.578 × 10^10^ L/mol for mAb 1E3 and 3.273 × 10^9^ L/mol for mAb 5H3 (Figure 3C).

### 3.4. Expression and Identification of Truncated Proteins

**Figure 4:**
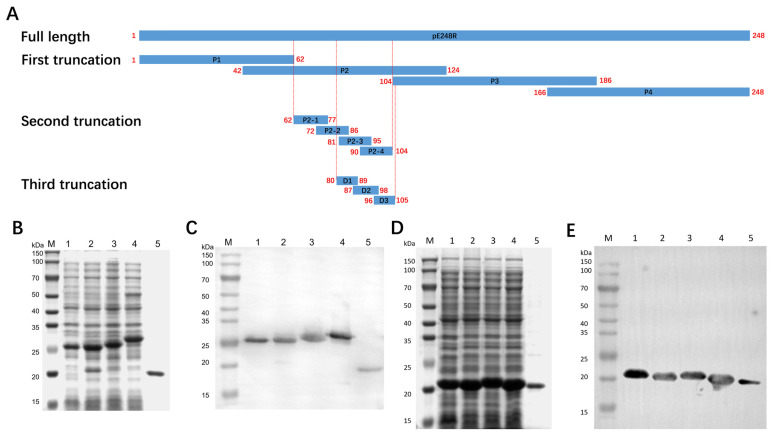
Expression and characterization of truncated proteins. (**A**) Schematic representation of the truncated pE248R proteins. (**B**,**C**) Expression and identification of the P1 to P4 truncated proteins. Lanes 1–4 represent the P1–P4 truncated proteins, and lane 5 corresponds to the pET-32a-expressed protein as control. (**D**,**E**) Expression and identification of the P2-1 to P2-4 truncated proteins. Lanes 1–4 represent the P2-1 to P2-4 truncated proteins, and lane 5 corresponds to the pET-32a-expressed protein as control.

Initially, the full-length pE248R protein was divided into four truncated fragments. The recombinant protein of each fragment has a molecular weight of approximately 25 kDa. The antigenic region identified thereafter was subsequently refined into four shorter segments (Figure 4A). All expressed truncations were confirmed by SDS-PAGE (Figure 4B,D), showing sizes consistent with theoretical predictions. Furthermore, each recombinant truncation exhibited specific reactivity with an anti-His monoclonal antibody (Figure 4C,E), validating their successful expression and antigenic integrity. In total, eight truncated recombinant proteins were successfully obtained for epitope mapping.

### 3.5. Antigenic Epitope Mapping and Identification of pE248R

**Figure 5:**
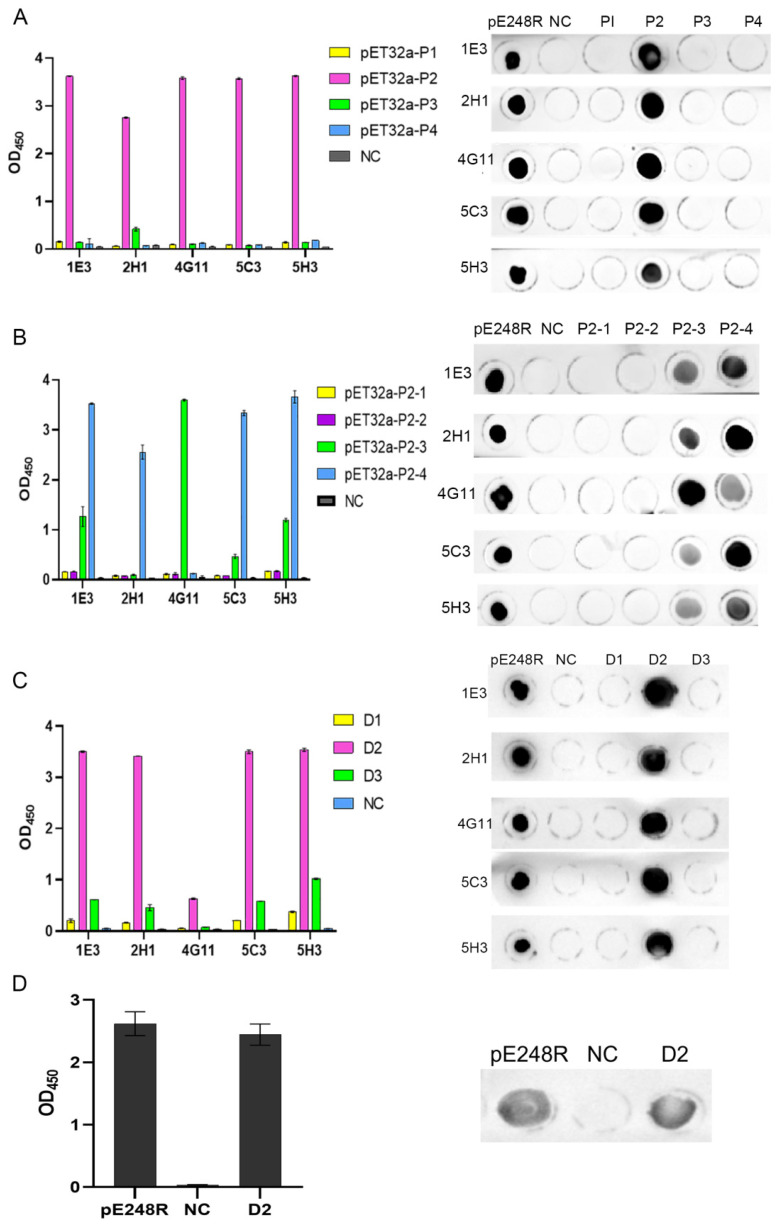
Antigenic epitope mapping of pE248R. (**A**) Detection of the reactivity of P1–P4 polypeptide fragments with monoclonal antibodies. (**B**) Detection of the reactivity of P2-1–P2-4 polypeptide fragments with monoclonal antibodies. (**C**) Detection of the reactivity of D1–D3 polypeptide fragments with monoclonal antibodies. (**D**) Detection of the reactivity of the D2 epitope peptide with ASFV-positive swine serum. The negative control was PBS.

To map the epitope region of the pE248R protein recognized by the monoclonal antibodies, we evaluated the binding activity of five mAbs (1E3, 2H1, 4G11, 5C3, and 5H3) against a set of overlapping polypeptide fragments (Figure 5). Initial screening with four polypeptides—P1 (aa 1–62), P2 (aa 42–124), P3 (aa 104–186), and P4 (aa 166–248)—revealed that only P2 bound to all five mAbs, while P1, P3, and P4 showed no reactivity (Figure 5A). Based on this finding, four finer polypeptides spanning the P2 region were synthesized. These further tests indicated that mAbs 1E3, 2H1, 4G11, 5C3, and 5H3 specifically recognized both P2-3 (aa 81–95) and P2-4 (aa 90–104) (Figure 5B), localizing the epitope within residues 81–104.

Subsequent fine mapping using peptide ELISA and dot-blot assays identified three linear antigenic epitopes recognized by the five mAbs. Among these, the peptide D2 (aa 87–98) was consistently recognized by all five mAbs (Figure 5C). Moreover, both the D2 epitope peptide and the full-length pE248R recombinant protein reacted specifically with ASFV-positive swine serum (Figure 5D), and the results from both assays were consistent. Collectively, these data define ^87^QEVALTQWMDAG^98^ as a linear B-cell epitope of the ASFV pE248R protein recognized by the screened monoclonal antibodies.

### 3.6. Conservation Analysis and Spatial Localization of the Linear B-Cell Epitope of pE248R Protein

**Figure 6:**
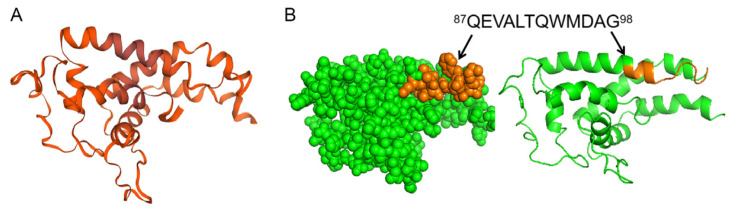
Localization of the linear B-cell epitope of pE248R protein. (**A**) The ASFV pE248R protein was modeled using SWISS-MODEL (https://swissmodel.expasy.org/) to generate the 3D structure. (**B**) The position of the linear B cell epitope (^87^QEVALTQWMDAG^98^, highlighted in orange) within the pE248R protein structure.

The conservation of the linear B-cell epitope of pE248R protein identified above were analyzed in 40 isolates generated in Table S1. According to the result of clustal W alignment, the conservation of ^87^QEVALTQWMDAG^98^ in epidemic strains were 100% (Figure S1). The three-dimensional structure of the pE248R protein was generated by homology modeling using SWISS-MODEL (Figure 6A). The identified linear epitope sequence was mapped onto the resulting protein model and visualized using PyMOL. As shown in Figure 6B, the epitope (^87^QEVALTQWMDAG^98^, highlighted in orange) is situated on the solvent-accessible surface of the pE248R protein (shown in green), consistent with its recognition by monoclonal antibodies.

## 4. Discussion

To date, extensive research has been conducted on linear B-cell epitopes of ASFV viral proteins, including p72 [12,15,32], p54 [13,33], and p30 [14,34], among others. Compared to these OIE recommended antigens, there is currently no evidence indicating that the pE248R protein possesses distinct advantages. However, only one live attenuated gene-deleted vaccine, ASFV-G-ΔI177L, has been approved for limited use in Vietnam [35]. Given that existing studies on immunodominant ASFV antigens have not yet achieved complete protection against viral infection, exploring the immunogenic functions of other viral proteins is of critical importance. Studies have shown that the candidate vaccine fused with pE248R and other ASFV antigens successfully induced strong ASFV-specific humoral and cellular immunity in pigs [27]. And given its role in ASFV replication, infection, and immune evasion, the investigation of B-cell epitopes on pE248R holds considerable significance for its development as a vaccine candidate. Furthermore, from a vaccine design perspective, the diversity of B-cell epitopes offers significant benefits—it enables the rational combination of multiple effective linear epitopes to develop multivalent vaccines. Such vaccines can simultaneously elicit a potent and broad-spectrum immune response targeting multiple epitopes, thereby enhancing immunogenic potency and reducing the risk of viral escape through mutation. From an ASFV diagnostic standpoint, pE248R—a key protein involved in the early stages of viral infection [19]—may exhibit unique diagnostic characteristics due to its distinct functional sites. Certainly, subsequent studies will require extensive experimentation to validate the sensitivity and specificity of the identified B-cell epitope, as well as its conservation across diverse viral genotypes. Nonetheless, our findings hold the potential to complement the existing frameworks for both ASFV vaccines and diagnostics.

A recent study, utilizing bioinformatic predictions and immunogenicity analyses, identified three linear B-cell epitopes of pE248R: ^121^NCVSSLSGMNVLVVKG^136^, ^138^GNIVENATQKQSQQIISNCLQGSKQAIDTTTG^169^, and ^158^QGSKQAIDTTTGITNTVNQYSHYTSKNF^185^ [15]. In contrast, our approach relied on epitope mapping using monoclonal antibodies for B-cell epitope identification. Among the diverse techniques currently available for B-cell epitope characterization—including peptide microarray technology, synthetic peptide approaches, X-ray crystallography, phage display peptide libraries, prokaryotic expression display systems, yeast surface display systems, and ELISA—the method employed in our study is recognized for its high reliability [15]. Furthermore, analysis of the reported findings suggests that two of the three epitope sequences identified in the aforementioned study may have the potential to be further truncated. Additionally, it is highly probable that a single viral protein contains multiple linear B-cell epitopes. Examination of the predicted protein structure of pE248R indicates that regions exposed on the molecular surface, exhibiting hydrophilicity and structural flexibility, are likely accessible for recognition by immune cells; such regions are often “hotspots” for linear epitopes.

Owing to the stringent biosafety requirements for working with live ASFV, our study successfully identified a linear B-cell epitope but could not proceed to functional validation involving the infectious virus. Consequently, the evidence supporting the biological relevance of this epitope remains preliminary. To strengthen these findings, future work should involve collaborations with specialized containment laboratories capable of handling live ASFV. Key functional assays would include virus neutralization tests, competition assays with field serum samples, and confirmation of the epitope’s surface accessibility on the native virus. Furthermore, integrating this epitope into the broader landscape of ASFV epitope research will be crucial for informing the rational design of effective multivalent vaccines.

In this study, the recombinant pE248R protein was expressed and purified using a prokaryotic expression system. The purified recombinant protein was employed as an immunogen to inoculate mice, leading to the generation of mAbs against pE248R via cell fusion. Following screening and characterization, five distinct monoclonal hybridoma cell lines were successfully established. A linear B-cell epitope within the ASFV pE248R protein (^87^QEVALTQWMDAG^98^) was subsequently identified. Collectively, these findings advance our understanding of the ASFV pE248R protein’s function, thereby providing a foundation for elucidating viral infection and immune evasion mechanisms, particularly contributing to the construction of a comprehensive B-cell epitope map for ASFV immunogens.

## Figures and Tables

**Table 1:** Primers for truncating gene fragments of E248R.

Primers	Sequence (5′–3′)	Position (Amino Acid)
P1-F	CGCGGATCCATGGGCGGCTCTACCTC	1–62 aa
P1-R	CCGCTCGAGTTAACAGGAGGTGTTCAGAGA	
P2-F	CGCGGATCCATGGGCGACGGCAACATCC	42–124 aa
P2-R	CCGCTCGAGTTAAGACACGCAGTTCTGGAT	
P3-F	CGCGGATCCATGACAGACATTGAGGAGAAC	104–186 aa
P3-R	CCGCTCGAGTTAAAAGAAATTCTTGCTGGT	
P4-F	CGCGGATCCATGACCACCACCGGCATCA	166–248 aa
P4-R	CCGCTCGAGTTAGCTCACGGCGGCGTT	
P2-1-F	CGCGGATCCATGGTGCAGAAGCATGTGA	62–77 aa
P2-1-R	CCGCTCGAGTTAGCTCAGATTGGTGATAAA	
P2-2-F	CGCGGATCCATGTTTATCACCAATCTGAGC	72–86 aa
P2-2-R	CCGCTCGAGTTAGTCCTTCAGGTTCTGT	
P2-3-F	CGCGGATCCATGACACAGAACCTGAAGG	81–95 aa
P2-3-R	CCGCTCGAGTTACATCCACTGGGTCAG	
P2-4-F	CGCGGATCCATGGCCCTGACCCAGTG	90–104 aa
P2-4-R	CCGCTCGAGTTATTTCTGATCGTGGGTGC	

**Table 2:** The truncated peptide sequences of pE248R protein.

Name	Sequence
D1	^80^ITQNLKDQEV^89^
D2	^87^QEVALTQWMDAG^98^
D3	^96^DAGTHDQKTD^105^

## Data Availability

The original contributions presented in this study are included in this article. Further inquiries can be directed to the corresponding authors.

## Supplementary Materials

The following supporting information can be downloaded at: https://www.mdpi.com/article/10.3390/microorganisms13112616/s1, Figure S1: Conservation analysis of the linear B-cell epitope ^87^QEVALTQWMDAG^98^ of pE248R protein; Table S1: ASFV isolates involved in multiple sequence alignment in this study.
