# Supplementary material for: Identification of a Linear B-Cell Epitope in the African Swine Fever Virus pE248R Protein Targeted by Monoclonal Antibodies
Source: Microorganisms. 2025 Nov 18;13(11):2616. doi: 10.3390/microorganisms13112616 (PMC12654683; doi:10.3390/microorganisms13112616)
Supplement: Supplementary file 1 [file microorganisms-13-02616-s001.zip › Table S1.pdf]

Supplementary table 1. ASFV isolates involved in multiple sequence alignment in this study

No.	Isolate	Country	Genotype	Genome Accession	Protein ID of pE248R
1	BA71V			NC_001659	NP_042824.1
2	OURT88/3	Portugal	I	NC_044957	YP_009703738.1
3	Nu1979	Italy	I	MW723481	UCX48638.1
4	CAM1994/1	Cameroon	I	OR387520	WNK22069.1
5	LO2018	Italy	I	MW647171	UFQ11361.1
6	K49	Zaire	I	MZ202520	QZK26688
7	19155_WB	Italy	I	OP312970	WFD51063.1
8	Pig/SD/DY-I/2021	China	I	MZ945537	UEN73298.1
9	Pig/HeN/ZZ-P1/2021	China	I	MZ945536	UEN73140.1
10	Georgia_2007/1	Georgia	II	NC_044959	YP_009927255.1
11	MAD/01/1998	Madagascar	II	OP781309	WLE30092.1
12	TAN/01/2001	Tanzania	II	OQ434234	WLE28997.1
13	MAL/04/2001	Malawi	II	OP781310	WLE30276.1
14	MOZ/01/2005	South Africa	II	OP781313	WLE30827.1
15	Stavropol_01/08	Russia	II	PQ672299	XNX60817.1
16	ZIM/2015	Zimbabwe	II	OP781311	WLE30459.1
17	Lv17/WB/Rie1i	Latvia	II	OR863252	WPS70932.1
18	RSA/08/2019	South Africa	II	OP781312	WLE30644.1
19	MAL/19/Karonga	Malawi	II	MW856068	QXP50090.1
20	Nigeria-RV502	Nigeria	II	OP672342	WFV29848.1
21	RWA/Rwamagana/2021	Rwanda	II	PQ375363	XOD29792.1
22	SY18	China	II	MH766894	WKD79638.1
23	China/2018/AnhuiXCGQ	China	II	MK128995	AYW34102.1
24	Lv17/WB/Rie1i	Spain	II	OR863252	WPS70932.1
25	Kyiv/2016/131	Ukraine	II	MN194591	QED21744.1
26	POL/2015/Podlaskie	Poland	II	MH681419	XBC37175.1
27	Korea/pig/Yeoncheon1/ 2019	Korea	II	MW049116	XBC37175.1
28	Pig/Jiangsu/LG/2021	China	I & II	OQ504956	WFS78587.1
29	Pig/Inner Mongolia/DQDM/2022	China	I & II	OQ504955	WFS78415.1
30	RSA_W1_1999	South Africa	IV	MN641876	QST87212.1
31	Spec_57	South Africa	VIII	MN394630	QGM12943.2
32	RWA/Musanze/2023	Tanzania	IX	PQ375362	XOD29616.1
33	RSA_2_2004	South Africa	XX	MN641877	QST87050.1
34	RSA_2_2008	South Africa	XXII	MN336500	QGM12869.2
35	Kaliningrad_18/WB-9734	Russia	unannotated	OM966721	UVH36014.1
36	Germany 2020/1	Germany	unannotated	LR899193	CAD7112642.1
37	Belgium 2018/1	Belgium	unannotated	LR536725	VFV48065.1

38	Timor-Leste/2019/1	Australia	unannotated	MW396979	QTE18833.1
39	Korea/CW714/2020	Korea	unannotated	OR162436	WMM66617.1
40	Korea/PC1432/2021	Korea	unannotated	OR180305	WMQ58430.1
