# Supplementary figures and images for: Identification of a Linear B-Cell Epitope in the African Swine Fever Virus pE248R Protein Targeted by Monoclonal Antibodies
Source: Microorganisms. 2025 Nov 18;13(11):2616. doi: 10.3390/microorganisms13112616 (PMC12654683; doi:10.3390/microorganisms13112616)

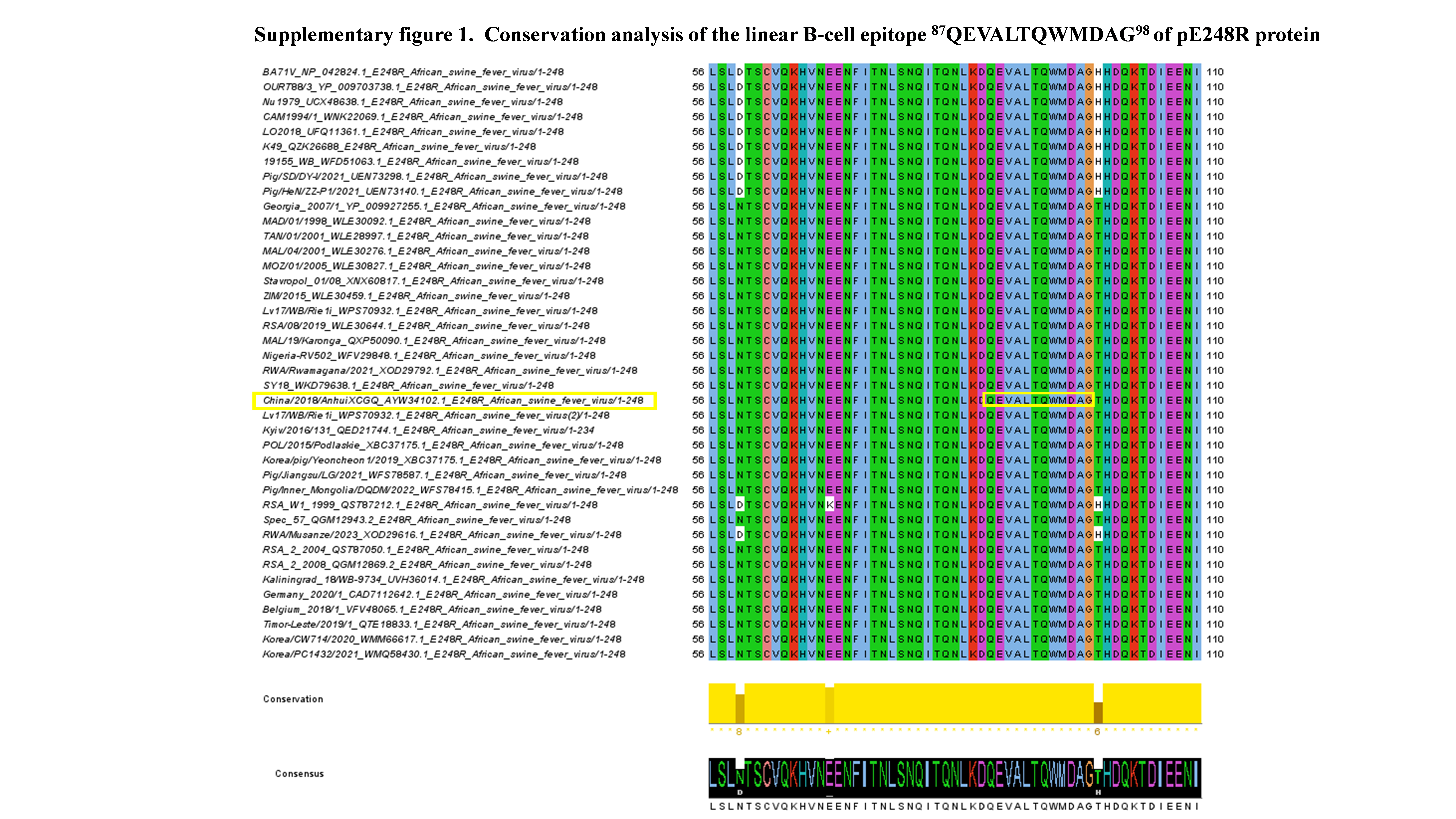

Supplement: Supplementary file 1 [file microorganisms-13-02616-s001.zip › Figure S1.tif]
